# Assessment of antiphospholipid antibodies profiles based on severity of COVID-19 pneumonia

**DOI:** 10.11604/pamj.2022.42.110.33020

**Published:** 2022-06-09

**Authors:** Amène Ben Bnina, Refka Ben Dhia, Sahar Gnaba, Alaa Annabi, Syrine Chouchane, Walid Naija, Houyem Said, Abderraouf Oueslati, Amina Bouatay

**Affiliations:** 1 Hematology Laboratory, Sahloul Teaching Hospital, 4054, Sousse, Tunisia,; 2 Department of Anesthesia and Intensive Care, Sahloul Teaching Hospital, 4054, Sousse, Tunisia,; 3 Department of Prevention and Care Safety, Sahloul Teaching Hospital, 4054, Sousse, Tunisia

**Keywords:** Antibodies, antiphospholipid, COVID-19, thrombosis, lupus anticoagulant

## Abstract

**Introduction:**

thrombotic events are the most severe complications of the coronavirus disease 2019 (COVID-19). It is known that anti-phospholipid antibodies (APL) could be involved in thrombosis mechanism. Thus, APL profiles were studied particularly in patients with severe and critical COVID-19, and their clinical impact.

**Methods:**

a retrospective study of 54 COVID-19 hospitalized patients (34 in intensive care unit (ICU) and 20 in non-ICU) was conducted. These COVID-19 patients were tested for the presence of LAC (lupus anticoagulant) using the ACLTOP750®, anti-cardiolipine (ACL) and anti-β2glycoprotéine I (anti-β2GPI) IgG/IgM/IgA by enzyme-linked immunosorbent assay (ELISA). IgA isotype was tested in only 25 patients.

**Results:**

anti-phospholipid antibodies were present in 74.1% of tested patients. LAC positivity was the highest (60.8%) among all patients, followed by IgM aCL (18.5%) and IgM anti-β2GPI (14.8%). Besides, LAC and anti-β2GPI IgA were the most predominant APL regarding the 25 patients tested for IgA isotype (52% and 24% respectively). Nine patients had thrombotic events, among them 6 were positive in APL and 5 were positive in LAC. However, there was any significant association between APL positivity or titers and thrombosis. There was also no significant difference between the two COVID-19 groups regarding APL profiles.

**Conclusion:**

given the relatively high frequency of APL and especially LAC, and given the multitude of thrombotic risk factors in these severely and critically ill COVID-19 patients, a prophylactic anticoagulation remains essential.

## Introduction

Thromboembolic events are out of the most severe complications in the course of the infection caused by severe acute respiratory syndrome coronavirus 2 (SARS-CoV-2). These thrombotic events lead to the high mortality rates, as these complications may be unrecognized or tardily diagnosed [1]. The main mechanism for developing these thrombotic complications remains unclear and is still debated by authors [2,3]. On the other hand, antiphospholipid syndrome (APS) is an autoimmune disease with a prothrombotic state associated with multiple arterial and venous thromboembolisms. Antiphospholipid syndrome is characterized by persistent antiphospholipid antibodies (APL). Laboratory criteria of APS are based on screening for anticardiolipin (ACL), anti-β2 glycoprotein 1 (anti-β2GPI) and lupus anticoagulant (LAC) antibodies [4]. Interestingly, high frequency of APL in patients with Coronavirus Disease 2019 (COVID-19) has been noticed in many studies [5-8]. Despite this association, clinical impact of these APL on thromboembolic events is not yet established [8]. Therefore, we aimed in our study, to test the presence of APL antibodies in intensive care-unit (ICU) and non-ICU hospitalized COVID-19 patients. We also aimed to evaluate the possible association of APL antibodies with thrombotic incidents and severity of the disease in these patients.

## Methods

**Study design and sampling:** in our cross-sectional study, a total number of 54 patients diagnosed with SARS-CoV-2 infections were included. Among them, 34 patients were critically ill and hospitalized in ICU, and 20 patients were in severe condition and hospitalized in non-ICU. Sera were collected from Sahloul university hospital in the center of Tunisia between January 2021 and April 2021. Included patients were all consecutive patients older than 18 years with confirmed SARS-CoV-2 infection and who required hospitalization in ICU or non ICU in the period of the study. The COVID-19 infection was confirmed by the detection of SARS-CoV-2 genome in nasopharyngeal swab samples. Our non-inclusion criteria were pregnancy, active cancer and incomplete data in medical files.

**Ethical considerations:** the study was approved by the local ethics committee of Sahloul University Teaching Hospital. All data and patients´ identities were processed with strict confidentiality.

**Data collection:** demographic data (age, gender, and underlying diseases), clinical, radiological and biological findings (D-dimer, fibrinogen, C-reactive protein (CRP), white cell count and platelet count) were collected either by consulting medical files or by referring to electronic hospital medical records.

**Anti-phospholipid antibodies detection:** fifty-four patients were tested for the positivity of APL antibodies during the active COVID-19 infection. ACL IgG, ACL IgM, anti-β2GPI IgG and anti-β2GPI IgM were measured in all of the 54 patients. However, ACL IgA and anti-β2GPI IgA were measured in only 25 patients (5 ICU hospitalized patients and 20 non-ICU hospitalized patients). Lupus anticoagulant was measured in 51 patients. ACL and anti-β2GPI IgG/IgM/IgA and IgG/IgM/IgA were measured by an enzyme-linked immunosorbent assay (ELISA) using the commercial ELISA kit of ORGENTEC® (Orgentec Diagnostika®, Mainz, Germany). The tests were done according to the manufacturer´s instructions. Anti-β2GPI IgG, IgM and IgA were considered positive at a cut-off value of ≥ 8 U/ml. Anticardiolipin IgG, IgM and IgA were considered positive at cut-off values of ≥ 10 GPL-U/ml, ≥ 7 MPL-U/ml and ≥ 10 APL-U/ml respectively. The presence of LAC antibodies were studied using the dilute Russell Viper Venom Time (dRVVT) and silica clotting time (SCT) tests (HemosIL dRVVT and HemosIL SCT, Instrumentation Laboratory, Milan, Italy). If SCT screen and/or dRVVT screen tests were prolonged comparing to the mean SCT/dRVVT screen normal range, patients were tested for confirmations with SCT/dRVVT confirm reagents containing an additional amount of phospholipid. Lupus anticoagulant were considered positive if normalized SCT ratio was higher than 1.16 and/or normalized dRVVT ratio was higher than 1.2. The normalized SCT and dRVVT ratios were calculated by dividing the corresponding test screen ratio on the confirm ratio. The screen/confirm ratios were calculated by dividing the patient clot time on the mean normal range clot time. All coagulation tests were performed using the ACLTOP® family system (instrumentation laboratory, Milan, Italy).

**Statistical analysis:** all statistical analyses were conducted using the software Statistical Package for the Social Sciences (SPSS) version 23.0. Results were described using mean and standard deviation (SD) for continuous variables, count and percentage for categorical variables. To assess the association between APL and thrombosis and between APL and severity of COVID-19, Student´s test was used for continuous variables, while Chi-square test or Fisher's exact test (when the theoretical number of patients was <5) were used for categorical variables. A p-value of less than 0.05 was considered as statistically significant.

## Results

**Patients´ characteristics:** the 54 patients enrolled in our study had a mean age of 62.8 ± 13.4 (20-89) years and were predominantly male (61%) with a sex-ratio of 1.57. The most common underlying diseases were; hypertension (37%) and diabetes (31.5%). Only 3 patients had a past medical history of thrombosis. On admission, all patients had oxygen saturation levels below 94% and were mechanically ventilated. Among them, seven patients (12.7%) required intubation during hospitalization. Mean levels of APACHE II (Acute Physiologic Assessment and Chronic Health Evaluation II) and SOFA (Sequential Organ Failure Assessment) score were 12.9 ± 8.4 (5-45) and 5.6 ± 3.2 [2-14] respectively in ICU patients. Fifty-one of the 54 COVID-19 patients showed typical chest imaging features compatible with COVID-19 pneumonia. During the hospital stay, nine patients (16.7%) developed a thrombotic incident. Among them, two patients had a past medical history of venous thrombosis. The most common encountered thrombotic event was ischemic stroke (4 patients), followed by pulmonary embolism (three patients). Furthermore, two patients had arterial thrombosis. The first one had a mesenteric ischemia, while the other one developed a thrombosis of the right internal iliac artery. The demographic and clinical characteristics of studied patients are shown in Table 1.

**Table 1:** characteristics of ICU and non-ICU COVID-19 patients

	Total	ICU		Non-ICU	P-value
**Number of patients**	N=54	N=34		N=20	-
**Age (years)**	62.8±13.4 (20-89)	61.8±13.9 (20-84)		64.3±12.7 (38-89)	0.513
**Male/female ratio**	33/12 (1.57)	22/12(1.83)		11/9 (1.22)	0.480
**Medical history**					
Chronic obstructive pulmonary disease	2 (3.7%)	1(2.9%)		1(5%)	1.000
Asthma	0	0		0	**-**
Hypertension	20 (37%)	10 (29.4%)		10 (50%)	0.130
Diabetes	17 (31.5%)	9 (26.5%)		8(40%)	0.301
Cardiovascular disease	8 (14.8%)	5 (14.7%)		3 (15%)	1.000
History of thromboembolic event	3 (5.6%)	3 (8.8%)		0	0.287
**Clinical symptoms on admission**					
Fever	28 (51.9)	17 (50)		11 (55)	0.723
Cough	30 (55.6)	18 (52.9)		12 (60)	0.614
Dyspnea	39 (72.2)	20 (58.8)		19 (95)	0.004
Headache	8 (14.8)	5 (14.7)		3 (15)	1.000
Acute respiratory distress syndrome	9 (16.6)	9 (26.5)		0	0.019
Thrombosis	9 (16.6)	8 (23.5)		1 (5)	0.131
**Laboratory findings at the time of the APL measurement**					
White cell count (x103/mm3)	14.85 ± 8.94	18.3 ± 9.5		9 ± 3.24	≤0.0001
Platelet count (x103/mm3)	282 ± 136	283 ± 137		281 ± 136	0.975
D-dimer (μg/ml)	7.12 ± 14.86	8.48 ± 13.33		4.9 ± 17.22	0.416
Fibrinogen (g/L)	4.62 ± 1.31	4.82 ± 1.32		3.61 ± 0.55	0.002
C-reactive protein (mg/L)	114 ± 118	158 ± 125		36.5 ± 40.3	≤0.0001
**Clinical outcome**					
In-hospital death n (%)	30 (55.6)	28 (82.4)		2 (10)	≤0.0001

Abbreviations: ICU: intensive care unit

**Prevalence of APL antibodies in COVID-19 patients:** overall, APL antibodies were present in 74.1% (40/54) of tested plasma samples. Lupus anticoagulant positivity was the highest (60.8%) among all patients, followed by IgM ACL and IgM anti-β2GPI encountered in 18.5% and 14.8% patients respectively. Besides, among the 25 patients who were tested for IgA, six were positive in anti-β2GPI IgA. None of the patients was positive in ACL IgA. Among the 40 APL positive patients, 25 patients were critically ill patients and hospitalized in ICU, and 15 were non-ICU hospitalized patients. Antiphospholipid frequencies in ICU patients and non-ICU patients were summarized in Table 2. However, there was no significant difference between the two groups ICU and non-ICU patients, on the basis of APL positivity (Table 2). Regarding multiple APLs, nine patients had at least three positive APL antibodies simultaneously. The association of LAC + IgM antiβ2GPI + IgM ACL was the most observed profile (three patients) (Table 2). We also analyzed, the association between APL positivity and thrombosis. Lupus anticoagulant was frequent in patients who developed thrombotic complications (5/9; 55.5%). Six patients among the nine who developed a thrombotic event had positive APL profiles. Three patients had only one positive APL antibody. The first one had only positive IgA anti-β2GPI. The two other patients had only positive LAC. Moreover, two patients had an association of two positive APL (LAC+ IgM ACL and IgG ACL + IgG anti-β2GPI). The last patient had a triple positivity (LAC + IgM ACL + IgM anti-β2GPI). Nonetheless, no significant association was observed between APL positivity and thrombosis (p=0.681). In fact, LAC positivity, ACL positivity and anti-β2GPI positivity were not associated with thrombosis (p=0.696, p=0.681 and p=1 respectively). Antiphospholipid titers were low to moderate. Anticardiolipin IgG titers were 14.1±4.6 (min=10, max=20) GPL U/ml. ACL IgM titers were 11±4.6 MPL U/ml (min=7.1, max=20). Anti-β2GPI IgG titers were 19.2±5.8 (min=9, max=26) U/ml. Anti-β2GPI IgM titers were 14.8±8.4 (min=8, max=34) U/ml. Anti-β2GPI IgA titers were 17.6±11 (min=8, max=36) U/ml. However, there was no significant association between APL titers and thrombotic events.

**Table 2:** frequency of APL in ICU COVID-19 and non - ICU patients.

Single or multiple Positive APL antibodies	All patients (N=54)	ICU patients (N=34)	Non-ICU patients (N=20)	P-value
**Any APL n (%)**	40/54 (74.1)	25/34 (73.5)	15/20 (75)	1
**ACL**				
IgG n/N (%)	6/54(11.1)	5/34 (14.7)	1/20 (5)	0.395
IgM n/N (%)	10/54(18.5)	8/34 (23.5)	2/20 (10)	0.291
IgA n/N (%)	0/25	0/5	0/20	
**Anti-β2GPI**				
IgG n/N (%)	6/54(11.1)	5/34 (14.7)	1/20 (5)	0.395
IgM n/N (%)	8/54(14.8)	5/34 (14.7)	3/20 (15)	1
IgA n/N (%)	6/25(24)	0/5 ()	6/14 (42.8)	0.289
**LAC n/N (%)**	31/51 (60.8)	20/32 (62.5)	11/19 (57.9)	0.774
**Multiple APLs (≥3) n/N (%)**	9/54 (16.6)	6/34 (17.6)	3/20 (15)	1
LAC + IgM anti-β2GPI + IgM ACL	3	2	1	-
LAC + IgG anti-β2GPI + IgM ACL	1	1	-	-
LAC + IgA anti-β2GPI + IgG ACL	1	-	1	-
LAC + IgG anti-β2GPI + ACL (IgM + IgG)	1	1	-	-
LAC + IgM anti-β2GPI + ACL (IgM + IgG)	1	-	-	-
LAC + anti-β2GPI (IgM + IgA) + IgM ACL	1	-	1	-
LAC + anti-β2GPI (IgM + IgG) + IgM ACL	1	1	-	-

Abbreviations: ICU: intensive care unit, ACL: anticardiolipin antibodies, anti-β2GPI: antibeta2-glycoprotein I antibodies, LAC: Lupus anticoagulant

## Discussion

COVID-19 was particularly accompanied by coagulopathy, hemostatic changes and consequently thrombotic events [9]. It is known that APL antibodies could be involved in the pathways responsible for thrombosis [4,10]. Thus, in this study, we aimed to test the presence of APL antibodies in ICU and non-ICU COVID-19 infected patients and its impact on thrombosis occurrence. We have found APL in 74.1% of hospitalized COVID-19 patients. Among the critically ill COVID-19 patients, 73.5% were positive in APL, 62.5%, 32.4% and 29.4% were especially positive in LAC, ACL and anti-β2-GPI respectively. For the group of COVID-19 non-ICU hospitalized patients, 75% were positive in APL and 57.9%, 15% and 20% were especially positive in LAC, ACL and anti-β2-GPI. Regarding ACL and anti-β2-GPI isotypes, IgM was the most frequent isotype. Similarly, in a review of 23 studies, in which 250 COVID-19 patients were tested for APL, 64% were positive in LAC. Like our study, IgM was also the most frequent isotype of ACL and anti-β2-GPI antibodies. In the same review, ACL and anti-β2-GPI were positive in 9% and 13% respectively which is less frequent than ACL and anti-β2-GPI positivity in our study [3]. In the same way, Harzallah *et al*. [5], Devreese *et al*. [6] and Najim *et al*. [7] have found also high frequency of LAC positivity. However, they found low frequency of ACL and anti β2-GPI positivity in their critically ill COVID-19 patients. In another study of 104 non-ICU COVID-19 patients, it has been found that 47.1% were positive in at least one APL. Among these non-ICU patients, LAC was positive in 39.6% of patients (Table 3) [11]. Thus, high frequency of LAC positivity is the most common finding in hospitalized COVID-19 patients especially critically ill patients. However, some studies focused on the fact that high levels of CRP in critically ill patients can interfere with the methodology of phospholipid-dependent coagulation tests and induce false positive LAC [6]. Overall, the proportion of APL antibodies in COVID-19 hospitalized patients was much higher than the percentage of APL in the general population. In fact, in healthy young population, it has been found that LAC and ACL antibodies were observed in 1 to 5%. In older population, APL (ACL and anti-β2-GPI antibodies) were found in up to 12% [12]. Indeed, viral infections can trigger the production of APL antibodies and sometimes the occurrence of thromboembolic events. The increase of APL after viral infection can be due to molecular mimicry, and cross-reacting antibodies between viral antigens and host tissue β2-GPI [13]. However, while APL in COVID-19 patients were much higher than in general population, these antibodies were far below than in APS patients according to Gatto *et al*. [14]. In this study, prevalence and titers of APL in COVID-19 patients were significantly less regarding APS patients, and were not associated with thrombosis [14]. Similarly, in our COVID-19 patients, there was no significant association between APL positivity and thrombosis. Likewise, many studies didn´t find any association between APL and thrombotic complications in ICU and non-ICU hospitalized COVID-19 patients [6-8,15-17]. Nevertheless, other studies have found a significant association between APL positivity and thrombotic complications [8,18,19]. Regardless of the association between APL and clinical outcome of severe and critical COVID-19 patients, the conclusions of many studies show an only transient increase of APL due to COVID-19 [8].

**Table 3:** frequency of APL in hospitalized COVID-19 patients in literature

Authors	Tested patients	APL
Harzallah *et al*.	56 ICU patients	45% LAC, 10% IgG/IgM ACL or IgG/IgM anti-β2GPI.
Devreese *et al*.	31 ICU patients	67.7% LAC, 74% any APL
Najim *et al*.	60 ICU patients	37% any APL, 35% LAC, 1,6% anti- β2-GPI
Le Joncour *et al*.	104 non-ICU patients	47.1% any APL, 39.6% LAC, 33.7% ACL, 8.7% anti-β2-GPI.
Present study	34 ICU and 20 non-ICU	**-ICU**: 73.5% any APL, 62.5% LAC, 32.4% ACL, 29.4% anti-β2-GPI. -**non-ICU:** 75% any APL, 57.9% LAC, 15% ACL, 20% anti-β2-GPI

Abbreviations: ICU: intensive care unit, ACL: anticardiolipin antibodies, anti-β2GPI: antibeta2-glycoprotein I antibodies, LAC: Lupus anticoagulant, APL: antiphospholipid antibodies

Then, in our study, titers and positivity of APL were neither associated with thrombosis, nor with severity of COVID-19. In fact, titers were low to moderate in both ICU and non-ICU hospitalized COVID-19 patients and frequency of APL were nearly similar in the two groups (73.5% for ICU and 75% for non-ICU patients). Like our study, Xiao *et al*. tested 66 critically ill and 13 non-critically ill COVID-19 patients. However, in this Chinese study it has been shown a huge difference between the two COVID-19 groups. There was a total absence of APLs in non-critically ill COVID-19 patients and 47% of critically ill patients positive in APL. APL titers were higher than our study [20]. This discrepancy of APL profiles and their clinical associations could be explained by the difference between the epidemiological characteristics of COVID-19 patients included and the different methods used for APL measurement. Regarding the 25 patients who were tested for APL IgA, LAC and IgA anti-β2-GPI were the most common types found in this COVID-19 population (52% and 24% respectively). Nearly the same result was noted in the study of Xiao *et al*. who tested COVID-19 patients for ACL and anti-β2-GPI. Similarly, IgA anti-β2-GPI was the predominant type of APL in this study (28.8%) [20]. In fact, IgA isotype is involved in mucosal immunity. The alteration of pulmonary and intestinal mucosae in COVID-19 can be probably accompanied by breakage of mucosal immunity tolerance which can explain the preferential increase of IgA APL in COVID-19 patients [20]. On the other hand, combined positivity of LAC, ACL, and anti-β2GPI (triple positive APL) and high titers of these antibodies have been shown to be high risk factors of thrombotic events [21-24]. However, APL profiles in many studies of COVID-19 patients had low risk profiles for thrombosis [6]. In the same way, in our population, we have found only 9/54 (16.6%) patients with triple positive APL and low to moderate titers of APL. Thus, APL antibodies found in our COVID-19 population are probably not pathogenic and rather transcient. In fact, viral infections can induce transciently non-pathogenic APL especially of IgM isotype [13,25,26].

Our study has some limitations. In addition to the small number of patients, positive APL samples need to be controlled 12 weeks later in order to test the presence of the APL syndrome in infected patients. These measurements could not be performed for two main reasons; first, 30 patients died during the hospitalization period and second, patients who survived were hospitalized for less than 12 weeks. Moreover, thrombotic events were only followed during hospitalization and patients were not followed up after being discharged.

## Conclusion

APL (especially LAC) proportion was highly prevalent in our COVID-19 patients with severe and critical conditions. However, there was no association between APL and thrombosis occurrence in these COVID-19 patients in our study. On this basis, further studies are needed to verify if these antibodies are persistent with clinical impact or rather transient and not pathogenic. Nevertheless, given the relatively high frequency of APL and especially LAC, and given the multitude of thrombotic risk factors in these severely and critically ill COVID-19 patients, a prophylactic anticoagulation remains essential.

### What is known about this topic

- Thrombotic events are severe complications in COVID-19;
- Anti-phospholipid antibodies can be involved in thrombosis mechanisms.

### What this study adds

- Anti-phospholipid antibodies especially Lupus anticoagulant are frequent in severely and critically ill COVID-19 patients;
- There is no significant association between APL positivity and thrombosis in COVID-19 patients;
- APL positivity and profiles does not depend on COVID-19 severity.

## References

[1] Lodigiani C, Iapichino G, Carenzo L, Cecconi M, Ferrazzi P, Sebastian T (2020). Venous and arterial thromboembolic complications in COVID-19 patients admitted to an academic hospital in Milan, Italy. Thromb Res.

[2] Hassett C, Gedansky A, Mays M, Uchino K (2020). Acute ischemic stroke and COVID-19. Cleve Clin J Med.

[3] Gkrouzman E, Barbhaiya M, Erkan D, Lockshin MD (2021). Reality check on antiphospholipid antibodies in COVID-19 associated coagulopathy. Arthritis Rheumatol.

[4] Devreese KMJ, Ortel TL, Pengo V, Laat B, the Subcommittee on lupus anticoagulant/antiphospholipid antibodies (2018). Laboratory criteria for antiphospholipid syndrome: communication from the SSC of the ISTH. J Thromb Haemost.

[5] Harzallah I, Debliquis A, Drenou B (2020). Lupus anticoagulant is frequent in patients with COVID-19. J Thromb Haemost.

[6] Devreese KM, Linskens EA, Benoit D, Peperstraete H (2020). Antiphospholipid antibodies in patients with COVID-19: a relevant observation. J Thromb Haemost.

[7] Najim M, Rahhal A, Khir F, Aljundi AH, Yousef SA, Ibrahim F (2021). Prevalence and clinical significance of antiphospholipid antibodies in patients with coronavirus disease 2019 admitted to intensive care units: a prospective observational study. Rheumatol Int.

[8] Stelzer M, Henes J, Saur S (2021). The role of antiphospholipid antibodies in COVID-19. Curr Rheumatol Rep.

[9] Connors JM, Levy JH (2020). COVID-19 and its implications for thrombosis and anticoagulation. Blood J Am Soc Hematol.

[10] Devreese KM (2012). Antiphospholipid antibodies: evaluation of the thrombotic risk. Thromb Res.

[11] Le Joncour A, Frere C, Martin-Toutain I, Gougis P, Ghillani-Dalbin P, Maalouf G (2021). Antiphospholipid antibodies and thrombotic events in COVID-19 patients hospitalized in medicine ward. Autoimmun Rev.

[12] Durcan L, Petri M (2017). Epidemiology of the antiphospholipid syndrome. Handbook of systemic autoimmune diseases. Elsevier Ltd.

[13] Abdel-Wahab N, Talathi S, Lopez-Olivo MA, Suarez-Almazor ME (2018). Risk of developing antiphospholipid antibodies following viral infection: a systematic review and meta-analysis. Lupus.

[14] Gatto M, Perricone C, Tonello M, Bistoni O, Cattelan AM, Bursi R (2020). Frequency and clinical correlates of antiphospholipid antibodies arising in patients with SARS-CoV-2 infection: findings from a multicentre study on 122 cases. Clin Exp Rheumatol.

[15] Borghi MO, Beltagy A, Garrafa E, Curreli D, Cecchini G, Bodio C (2020). Anti-Phospholipid Antibodies in COVID-19 are different from those detectable in the anti-phospholipid syndrome. Front Immunol.

[16] Galeano-Valle F, Oblitas CM, Ferreiro-Mazón MM, Alonso-Muñoz J, Del Toro-Cervera J, Di Natale M (2020). Antiphospholipid antibodies are not elevated in patients with severe COVID-19 pneumonia and venous thromboembolism. Thromb Res.

[17] Previtali G, Seghezzi M, Moioli V, Sonzogni A, Cerutti L, Marozzi R (2020). The pathogenesis of thromboembolic disease in covid-19 patients: could be a catastrophic antiphospholipid syndrome. Thromb Res.

[18] Hamadé A, Woehl B, Harzallah I, Talbot M, Tousch J, Jambert L (2021). Antiphospholipid antibodies in patients with coronavirus disease 2019 infection hospitalized in conventional unit. Blood Coagul Fibrinolysis.

[19] Reyes Gil M, Barouqa M, Szymanski J, Gonzalez-Lugo JD, Rahman S, Billett HH (2020). Assessment of lupus anticoagulant positivity in patients with coronavirus disease 2019 (COVID-19). JAMA Netw Open.

[20] Xiao M, Zhang Y, Zhang S, Qin X, Xia P, Cao W (2020). Antiphospholipid antibodies in critically Ill patients With COVID-19. Arthritis Rheumatol.

[21] Pengo V, Ruffatti A, Del Ross T, Tonello M, Cuffaro S, Hoxha A (2013). Confirmation of initial antiphospholipid antibody positivity depends on the antiphospholipid antibody profile. J Thromb Haemost.

[22] Pengo V, Ruffatti A, Legnani C, Gresele P, Barcellona D, Erba N (2010). Clinical course of high-risk patients diagnosed with antiphospholipid syndrome. J Thromb Haemost.

[23] Sciascia S, Murru V, Sanna G, Roccatello D, Khamashta MA, Bertolaccini ML (2012). Clinical accuracy for diagnosis of antiphospholipid syndrome in systemic lupus erythematosus: evaluation of 23 possible combinations of antiphospholipid antibody specificities. J Thromb Haemost.

[24] Mustonen P, Lehtonen KV, Javela K, Puurunen M (2014). Persistent antiphospholipid antibody (aPL) in asymptomatic carriers as a risk factor for future thrombotic events: a nationwide prospective study. Lupus.

[25] Vollmer O, Tacquard C, Dieudonné Y, Nespola B, Sattler L, Grunebaum L (2021). Follow-up of COVID-19 patients: LA is transient but other aPLs are persistent. Autoimmun Rev.

[26] Sène D, Piette JC, Cacoub P (2008). Antiphospholipid antibodies, antiphospholipid syndrome and infections. Autoimmun Rev.

